# Use of an endotracheal tube for surgical abortion complicated by a leiomyomatous uterus: a case report

**DOI:** 10.1186/s13256-017-1408-y

**Published:** 2017-08-25

**Authors:** Christy M. Boraas, Catherine A. Chappell, Colleen M. Krajewski

**Affiliations:** 1 Department of Obstetrics, Gynecology and Women’s Health, University of Minnesota Medical School, 606 24th Avenue S, Minneapolis, MN 55454 USA; 2 Department of Obstetrics, Gynecology and Reproductive Sciences, Magee-Womens Hospital of UPMC, University of Pittsburgh School of Medicine, 300 Halket Street, Pittsburgh, PA 15213 USA

**Keywords:** Abortion, Induced, Leiomyoma, Curettage, Case reports, Minimally invasive surgery

## Abstract

**Background:**

Abnormal uterine anatomy, especially leiomyomas, can significantly impact the difficulty and potential morbidity of surgical uterine evacuation. To avoid hysterotomy and/or hysterectomy, limited evidence exists to guide surgical uterine evacuation when pregnancy tissue is inaccessible with routine instruments.

**Case presentation:**

A 41-year-old G4P1021 African American woman at 14 4/7 weeks’ gestation was referred for surgical-induced abortion in the setting of an enlarged leiomyomatous uterus. Two large opposing leiomyomas at the internal cervical os rendered pregnancy tissue inaccessible with routine gynecologic surgical instruments. With ultrasound guidance, an endotracheal tube was connected to routine electric suction and utilized to complete uterine evacuation.

**Conclusions:**

With distorted or markedly enlarged uterine anatomy rendering pregnancy tissue inaccessible with routine surgical instruments, the minimally invasive use of an endotracheal tube may aid completion of uterine evacuation for surgical uterine evacuation.

## Background

Leiomyomas are benign smooth-muscle uterine tumors present in approximately one third of premenopausal women and can complicate both pregnancy and uterine evacuation by distorting uterine and cervical anatomy [1, 2]. In the presence of large leiomyomas, multiple case reports detail successful medical abortion as well as adjunctive treatment with misoprostol for cervical preparation, the use of a flexible cannula, and ultrasound guidance for surgical abortion [3–6]. Borgatta and Stubblefield suggest the use of other instruments to reach pregnancy tissue including nasogastric tubes, flexible cannulas telescoped through a rigid cannula, and endotracheal tubes (ETTs); however, no such reports exist in the published literature [7]. We present a case report using an ETT to complete surgical-induced abortion.

## Case presentation

A 41-year-old G4P1021 African American woman at 14 4/7 weeks’ gestation was referred for induced abortion in a hospital setting because of a large leiomyomatous uterus. She initially presented to an out-patient clinic for abortion care at 12 0/7 weeks. Restrictions prohibited hospital-based care for the patient in her home state and hospital-based care for medical management in any state. In consultation at our clinic, she described the presence of leiomyomas for 10 years with a 6-month history of increased menstrual flow but denied any abnormal bleeding, bulk, or pressure symptoms. She noticed a slight increase in abdominal girth at 9 to 10 weeks after last menses prompting pregnancy diagnosis. She denied any significant past medical or surgical history. Her obstetric history remotely included a missed abortion treated with medications, an uncomplicated vaginal delivery, and a spontaneous abortion. She reported a family history (multiple female relatives) of leiomyomas but no significant psychosocial history. Her preoperative examination was notable for a grossly enlarged, irregular, leiomyomatous uterus with a fundal height of 36 cm. Her cervix was easily visible on speculum examination, slightly displaced anteriorly and appeared normal.

Transabdominal ultrasonography revealed a single intrauterine pregnancy with embryonic cardiac activity at 14 4/7 weeks’ gestation just to the right of umbilicus. Multiple leiomyomas were seen throughout the uterus with four in the lower uterine segment alone. The largest were directly opposing each other at the level of the internal cervical os and measured 11.3 × 10.0 cm posteriorly and 5.7 × 6.5 cm anteriorly. The gestational sac could not be visualized on transvaginal ultrasonography given its distance from the external cervical os.

She received counseling about management options, including dilation and evacuation (D&E) and desired to avoid the potential morbidity of a major surgery (such as a hysterotomy abortion or hysterectomy). To evacuate the uterus for intrauterine fetal demise or termination of pregnancy at 14 weeks’ gestation, it is standard of care at our institution to offer D&E. Informed consent for D&E was obtained. Three synthetic osmotic dilators placed 1 day prior and misoprostol 400 mcg buccally 3 hours prior to scheduled D&E were used for cervical preparation. Her D&E procedure was performed utilizing deep sedation and a paracervical block. On bimanual examination, the external cervical os was dilated 1 to 2 cm and the internal os could not be palpated. Under ultrasound guidance, the cervical canal was dilated to 55 French with Pratt dilators. The entire length of the Pratt was needed to reach the most caudal portion of the gestational sac.

**Fig. 1:**
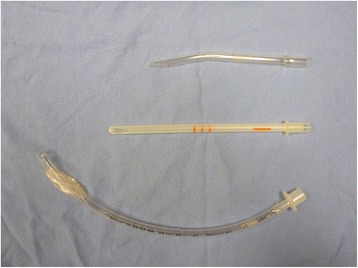
Comparison of 10 mm rigid curved suction cannula, 10 mm flexible suction cannula, and 8 mm endotracheal tube

Bierer and Sopher forceps were used to evacuate the majority of fetal tissue, however, the placenta was located beyond the reach of available forceps and suction cannulas. Two firm, curved, suction cannulas could not be used in telescoping fashion because the combination could not safely traverse the curvature of her cervical canal. A flexible suction cannula (functional length of 18 cm) was not long enough. An 8 mm ETT was obtained and the balloon occluder disconnected (Fig. 1). An 8 mm ETT (outer diameter 10.8 cm) was chosen because the outer diameter was similar to that of both the Sopher and Bierer forceps used in the case and we also chose it because it was expected to traverse the cervix and it was the longest (30 cm) of the available ETT sizes in the operating room.

The ETT was then attached to standard suction tubing for electric aspiration and inserted to the fundus of her uterus. Multiple passes were made until the uterine cavity had a gritty texture and a thin endometrial lining was noted on ultrasound. The uterine cavity sounded to a depth of 22 cm (measured with ETT) at the completion of the procedure. Surgical blood loss was minimal and there were no adverse events. Our patient was discharged the same day without complications. Given the distance she travelled to our clinic, follow-up was completed by telephone 1 week and again 1 month postoperatively; she reported minimal bleeding or discomfort postoperatively and was overall doing well. She was encouraged to establish gynecologic care for ongoing surveillance and potential management of symptoms related to her leiomyomatous uterus.

## Discussion

Leiomyomas in pregnancy are associated with multiple complications including spontaneous abortion, incomplete abortion, pain, fetal growth restriction, and hemorrhage. Expectant management of leiomyomas in pregnancy is common but can include medical and surgical management (myomectomy) dependent upon a patient’s characteristics [8]. With large leiomyomas, the uterine cavity may be markedly enlarged and/or distorted making surgical dilation and curettage or D&E particularly challenging in the case of early pregnancy failure, intrauterine fetal demise, or termination of pregnancy. Incomplete evacuation of pregnancy tissue in these settings can lead to infection, hemorrhage, and the potential for significant morbidity and mortality.

This case demonstrates the successful use of an ETT in completion of surgical abortion complicated by an enlarged leiomyomatous uterus. Our patient’s gestational sac and placental tissue were inaccessible to usual gynecologic instruments and an ETT was utilized for additional length, flexibility, and diameter of opening in the tube. The strengths of this approach include avoidance of major surgery and potential morbidity, the patient’s desire for a quick postoperative recovery, and future fertility is retained. The limitations of this approach include the inability to definitively manage the patient’s leiomyomatous uterus, however, our patient wanted to avoid hysterectomy.

General consensus is lacking on optimal surgical management of patients who require pregnancy interruption or treatment of early pregnancy loss with a markedly enlarged myomatous uterus. Dalton and Lebovic reported the use of a flexible cannula under ultrasound guidance to successfully bypass obstructing leiomyomas and evacuate a 9-week gestation in a 14-week-sized uterus [9]. No reports exist in the published literature guiding surgical management when pregnancy tissue is inaccessible to routine gynecologic instruments or detailing the use of an ETT for induced surgical abortion.

While the majority of surgical abortions occur safely in the out-patient clinic setting, some cases are optimally performed in an in-patient setting. Complex uterine anatomy, including distortion by multiple leiomyomas, is often a reason for referral to hospital-based care for patients seeking abortion care. While the use of an ETT may not be a feasible solution for all patients, when complex uterine anatomy is encountered, tools of other medical specialties may be helpful for surgical abortion and to avoid the increased risks and morbidity of hysterotomy abortion or hysterectomy. As with minimally invasive surgical techniques, the surgeon must be prepared to immediately proceed with hysterotomy or hysterectomy if the technique is unsuccessful or complications arise.

## Conclusions

This case report presents the use of an ETT to aid in uterine evacuation when pregnancy tissue was inaccessible to routine gynecologic instruments in the setting of complex, enlarged leiomyomatous uterus. Minimally invasive surgical techniques using novel instruments from other disciplines may be beneficial to increase a patient’s options and avoid the morbidity associated with more invasive surgical procedures.

## References

[1] Okolo S. Incidence, aetiology and epidemiology of uterine fibroids. Best Pract Res Clin Obstet Gynaecol. 2008;22(4):571–88. 10.1016/j.bpobgyn.2008.04.002 18534913

[2] Gibson E, Schreiber CA. When uterine leiomyomas complicate uterine evacuation. Contraception. 2010;82(6):486–8. 10.1016/j.contraception.2010.02.021 21074008

[3] Buckshee K, Dhond AJ. A new nonsurgical technique for termination of intrauterine pregnancy associated with large multiple uterine leiomyomas. Int J Gynaecol Obstet. 1992;37(4):297–9. 10.1016/0020-7292(92)90334-f 1350548

[4] Fenwick DK, Divers MJ. Medical pregnancy termination in the presence of a massive uterine fibroid. Br J Clin Pract. 1995;49(6):332–3. 8554964

[5] Creinin MD. Medically induced abortion in a woman with a large myomatous uterus. Am J Obstet Gynecol. 1996;175(5):1379–80. 10.1016/s0002-9378(96)70061-3 8942521

[6] Borgatta L, Sayegh R, Betstadt SJ, Stubblefield PG. Cervical obstruction complicating second-trimester abortion: treatment with misoprostol. Obstet Gynecol. 2009;113(2 Pt 2):548–50. 10.1097/AOG.0b013e318193bca7 19155952

[7] Borgatta L, Stubblefield PG. The challenging abortion. In: Paul M, Lichtenberg ES, Borgatta L, Grimes DA, Stubblefield PG, Creinin MD, editors. Management of unintended and abnormal pregnancy. 1st ed. West Sussex: Blackwell Publishing; 2009. p. 199–200.

[8] Vitale SC, Tropea A, Rossetti D, *et al.* Management of uterine leiomyomas in pregnancy: review of literature. Updates Surg. 2013;65(3):179–82. 10.1007/s13304-013-0198-z 23361218

[9] Dalton VK, Lebovic DI. Use of a flexible cannula to bypass an obstructing fibroid during a first-trimester surgical abortion. A case report. J Reprod Med. 2003;48(7):551–2. 12953330

